# Investigation of the risk factors to predict cytokine release syndrome in relapsed or refractory B-cell acute lymphoblastic leukemia patients receiving IL-6 knocking down anti-CD19 chimeric antigen receptor T-cell therapy

**DOI:** 10.3389/fimmu.2022.922212

**Published:** 2022-08-29

**Authors:** Wen-Jie Gong, Yan Qiu, Ming-Hao Li, Li-Yun Chen, Yan-Yan Li, Jing-Qiu Yu, Li-Qing Kang, Ai-Ning Sun, De-Pei Wu, Lei Yu, Sheng-Li Xue

**Affiliations:** ^1^ National Clinical Research Center for Hematologic Diseases, Jiangsu Institute of Hematology, The First Affiliated Hospital of Soochow University, Suzhou, China; ^2^ Institute of Blood and Marrow Transplantation, Collaborative Innovation Center of Hematology, Soochow University, Suzhou, China; ^3^ Research and Development Department, Shanghai UnicarTherapy Bio-Medicine Technology Co., Ltd., Shanghai, China; ^4^ Institute of Biomedical Engineering and Technology, Shanghai Engineering Research Center of Molecular Therapeutics and New Drug Development, School of Chemistry and Molecular Engineering, East China Normal University, Shanghai, China

**Keywords:** relapsed or refractory B-cell acute lymphoblastic leukemia, chimeric antigen receptor T-cell therapy, cytokine release syndrome, IL-6 knocking down, risk factors

## Abstract

CD19 chimeric antigen receptor-T (CAR-T) cell therapy has achieved remarkable results in patients with relapsed or refractory B-cell acute lymphoblastic leukemia (r/r B-ALL). However, the cytokine release syndrome (CRS) was presented in most patients as common toxicity and severe CRS (sCRS) characterized by the sharp increase in interleukin-6 (IL-6) could be life-threatening. We conducted a phase II clinical trial of ssCAR-T-19 cells, anti-CD19 CAR-T cells with shRNA targeting IL-6, in 61 patients with r/r B-ALL. This trial was registered at www.clinicaltrials.gov as #NCT03275493. Fifty-two patients achieved CR while nine patients were considered NR. The median duration of response (DOR) and overall survival (OS) were not reached (>50 months). CRS developed in 81.97% of patients, including 54.10% with grades 1 to 2 (grade 1, 31.15%; grade 2, 22.95%) and 27.87% with grades 3 to 4 (grade 3, 26.23%; grade 4, 1.64%). sCRS occurs earlier than mild CRS (mCRS). A multivariable analysis of baseline characteristics identified high bone marrow disease burden and poor genetic risk before infusion as independent risk factors for sCRS. After infusion, patients with sCRS exhibited larger expansion of ssCAR-T-19 cells, higher peak levels of IL-6, IL-10, and IFN-γ, and suffered more severe hematological and non-hematological toxicities compared with those with mCRS.

## Introduction

CD19 chimeric antigen receptor-T (CAR-T) cell therapy has achieved remarkable results in patients with relapsed or refractory B-cell acute lymphoblastic leukemia (r/r B-ALL) as a new treatment with a 54.5%–92.3% complete remission (CR) rate (1–3). Due to their outstanding clinical effectiveness, the Food and Drug Administration (FDA) and European Medicines Agency (EMA) have approved four CD19 CAR-T cell products to treat patients ≤25 years old with r/r B-ALL (Kymriah), adult patients with r/r B-ALL (Tecartus), patients with relapsed or refractory large B-cell lymphoma (r/r LBCL) (Kymriah, Yescarta and Breyanzi), patients with relapsed or refractory mantle-cell lymphoma (r/r MCL) (Tecartus) and patients with relapsed and refractory follicular lymphoma (Kymriah) (4–8).

However, the cytokine release syndrome (CRS) and immune effector cell associated neurotoxicity syndrome (ICANS) were presented in a majority of patients as common toxicities associated with CAR-T-cell therapy. CAR-T cells are “living drugs.” The development of CRS is directly related to *in vivo* CAR-T-cell expansion. The pharmacokinetics of CAR-T cells *in vivo* depend on several intrinsic and extrinsic factors, such as different costimulatory domains of CAR structures, tumor burden, and lymphodepletion regimen before CAR-T-cell infusion (9, 10). As CAR-T cells expand when interacting with the target tumor cells, massive cytokines including interleukin-6 (IL-6), interferon-γ (IFN-γ), monocyte chemoattractant protein 1 (MCP-1), and granulocyte-macrophage colony-stimulating factor (GM-CSF) were released by CAR-T cells and other immune cells. These cytokines can be associated with the clinical evidence of CRS (11–14). CRS often occurs within 14 days in patients receiving CAR-T therapy (15), while severe CRS (sCRS) could even occur rapidly within 1-2 days (16). The mild form of CRS (mCRS) often presented with flu-like symptoms such as fever, headache, and myalgia, whereas sCRS often presented with life-threatening symptoms like hypoxia, vasodilatory shock, capillary leak, and end-organ dysfunction, and even led to death (17). Although there is no temporal correlation between ICANS and CRS, neurotoxicity is confirmed to be caused by the mediated release of cytokines by CAR-T cells (18).

To manage CRS, tocilizumab or/and corticosteroids are often administered once CRS develops rapidly to a severe stage (higher than grade 3) in the clinic (19). However, tocilizumab is not effective in treating neurotoxicity caused by the CAR-T therapy because of its inability to pass the blood–brain barrier (BBB) and the influences of corticosteroids on CAR-T cells remain controversial (20). With currently passive treatment to manage sCRS, the incidence of sCRS is 8.3% to 43% (21–23), which is still the main obstacle to promoting CAR-T therapy.

Therefore, it is of great importance not only to choose safe and effective CAR-T products but also to investigate the risk factors to predict CRS early and alert the clinician to intervene timely before CRS deteriorates. Only in this way can we significantly reduce the risk of sCRS and bring more benefits to patients.

Thus, we conducted a phase II clinical trial applying IL-6 knocking down anti-CD19 CAR-T cells to products designed for safety (termed as ssCAR-T-19) for treating 61 patients with r/r B-ALL. The aim of this work was not only to assess the safety and efficacy of ssCAR-T-19 cells but also to explore the risk factors for potentially predicting the severity of CRS in patients accepting ssCAR-T-19 therapy.

## Materials and methods

### Study design and data collection

A phase II clinical trial (NCT03275493) was conducted to assess the safety and anti-tumor activity of CD19 CAR-T cells with IL-6 knockdown in patients with r/r B-ALL at our center (the First Affiliated Hospital of Soochow University). The data of enrolled 61 patients with r/r B-ALL receiving ssCAR-T-19 cell infusion from January 2017 to August 2020 were analyzed. The data cutoff date for the final analysis was October 26th. The electronic medical records of patients, including their clinical, laboratory, and treatment characteristics, were collected. Genetic risk stratification is shown in **Supplementary Table 1**. This study was conducted in accordance with the principles of the institutional review boards. Written informed consent was obtained from all patients.

### Inclusion and exclusion criteria

All the patients were enrolled according to the inclusion and exclusion criteria of the trial. Eligible patients had relapsed or refractory CD19^+^ B-ALL with an expected survival of ≥12 weeks but were ineligible for allogeneic hematopoietic stem cell transplantation (HSCT). Other inclusion criteria were (1) left ventricular ejection fractions ≥0.5 by echocardiography (2); ALT ≤3 times of ULN, or bilirubin <2.0 mg/dl (3); creatinine <2 mg/dl and less than 2.5 × normal for age (4); prothrombin time (PT) and activated partial thromboplastin time (APTT) <2 times of ULN (5); arterial oxygen saturation >92% (6); Karnofsky score ≥60; and (7) no history of combined chemotherapy in the recent 1 month and no immunotherapy in the recent 3 months.

Patients were excluded if they had (1) uncontrolled active infections (2); active hepatitis B or hepatitis C infection (3); HIV infection (4); history of myocardial infarction in the past 6 months, or history of severe arrhythmia (5); congenital immunodeficiency (6); pregnant or lactating women (7); history or presence of clinically relevant CNS pathology, such as epilepsy, generalized seizure disorder, paresis, aphasia, stroke, severe brain injuries, dementia, Parkinson’s disease, cerebellar disease, organic brain syndrome, or psychosis; and (8) previous treatment with any gene therapy products.

### Manufacturing of ssCAR-T-19 cells

The manufacturing process of ssCAR-T-19 cells is a multi-step process involving leukapheresis, separation, activation, transduction, expansion, and harvesting. Leukapheresis concentrates were obtained from patients at our center. T cells were separated using anti-CD3 magnetic beads (Miltenyi, Biotec, Bergisch-Gladbach, Germany), stimulated with anti-CD3/CD28 monoclonal antibodies (Miltenyi, Biotec, Bergisch-Gladbach, Germany), and transduced with a recombinant lentiviral vector. The structure of the recombinant lentiviral vectors is illustrated in **Supplementary Figure 1**. Except for an anti-CD19 murine single-chain variable fragment (scFv), a 4-1BB costimulatory moiety was encoded. The feature lies in the CD3zeta activation domain with an IL-6 shRNA element against IL-6. ssCAR-T-19 cells were cultured in AIM-V media (Gibco, NY, USA) supplemented with 10% autologous human serum, 100 IU/ml recombinant human IL-2 (PeproTech, Rocky Hill, USA), 5 ng/ml recombinant human IL-7 (PeproTech), and 5 ng/ml recombinant human IL-15 (PeproTech) for 12–14 days before infusion.

### Preparative lymphodepletion chemotherapy

All the patients accepted lymphodepletion chemotherapy before the infusion of ssCAR-T-19 cells to reduce tumor burden and endogenous lymphocytes. Of which, 60 patients were treated with fludarabine (Flu) (30 mg/m^2^) plus cyclophosphamide (Cy) (0.3 g/m^2^) based regimen and one patient received a FLAG regimen (5 days), including fludarabine (30 mg/m^2^), cytosine arabinoside (Ara-c) (1 g/m^2^), plus granulocyte-colony stimulating factor (G-CSF, 300 μg/d).

### SsCAR-T-19 cell infusion

Based on the adverse effects, tolerance and clinical design, all the patients received a dose of 5 × 10^6^/kg ssCAR-T-19 cells within 10–15 min. Two methods for fractionated dose were adopted according to infusion reactions (1): 10% of the total expected dose on day 1, 30% on day 2 and 60% on day 3; and (2) 40% of the total expected dose on day 1 and 60% on day 2, respectively. The vital signs of the patients, such as temperature, blood pressure, heart rate, respiration, and blood oxygen, were monitored closely before, during, and after the infusion until the patients were in stable condition. The infusion was stopped if a serious reaction occurred.

### Outcomes and endpoints

The primary endpoints were the overall response rate (ORR) and toxicities, particularly the occurrence of CRS. A response assessment was performed on day 28. CR was defined as <5% bone marrow blasts, no original lymphocytes in peripheral blood, and no recurrence within four weeks, regardless of cell count recovery. Partial remission (PR) was defined as 5%–20% bone marrow blasts. No remission (NR) was defined as >20% bone marrow blasts (NCCN Guideline Version 2021). CRS was graded on the basis of ASTCT CRS consensus grading (24) and the criteria at our center (**Supplementary Table 2**), and defined as mild CRS (mCRS) if graded 0–2 and severe CRS (sCRS) if graded 3–4. Analyses of complete blood counts, coagulation, hepatic function, renal function, and cardiac function were conducted to evaluate hematologic and non-hematologic side effects. The secondary endpoint was the duration of response (DOR), and overall survival (OS). DOR was defined as the time from the first complete remission after ssCAR-T019 infusion to a relapse or death without documented relapse. The OS was defined as the time from ssCAR-T-19 infusion to the date of death from any cause.

### Analysis of clinical laboratory parameters and serum biomarkers after ssCAR-T-19 cell infusion

Peripheral blood of the enrolled patients was collected after a ssCAR-T-19 cell infusion. The peak concentrations of cytokines, including IL-6, interleukin-10 (IL-10), IFN-γ, C-reactive protein (CRP), and ferritin were tested. The expansion and persistence of ssCAR-T-19 cells were detected by qRT–PCR.

### Statistical analysis and sample size

The study had approximately 90% power to distinguish between an active therapy with a rate of complete remission or complete remission with incomplete hematological recovery of 65% and a pre-specified, historical control rate of 44% or less with a one-sided α value of 0.025 (25, 26). Based on this hypothesis, the planned sample size was 59 patients. Statistical analysis was performed using SPSS 25.0 (SPSS Inc., Chicago, IL, USA) and Prism 6 (GraphPad Software Inc., San Diego, CA, USA). Numerical variables were described using median and interquartile spacing (IQR 25% and 75%). *P-*values were calculated using the t-test if data were normally distributed and the Mann–Whitney U test if not. Categorical variables were described by percentages and compared by the Chi-Square test and Fisher Exact test. Logistic regression analysis was used for univariate and multivariate analysis. Tables and graphs were designed using PowerPoint (Microsoft, Redmond, WA, USA), Prism 6 (GraphPad Software Inc.), and R-language. *P-*values <0.05 were considered as statistically significant. If not otherwise mentioned, results are presented as mean ± standard deviation (SD).

## Results

### Patients and treatment characteristics

Sixty-one patients with r/r B-ALL were included in the analysis. The median age was 32 years (IQR: 19.5, 45.5 years), and 30 (49.18%) patients were male and 31 (50.82%) were female. Thirty-four (55.74%) patients and their genetic risk were good, while 27 (44.26%) were poor. The median number of prior lines of therapy was 3 (IQR: 2, 4 prior lines) and the median number of relapses was 1 (IQR: 1, 1 relapses). Twelve patients (19.67%) had previously undergone allogeneic HSCT, while one patient (1.64%) had undergone autologous HSCT. The lymphodepletion regimens were given to all 61 patients before the ssCAR-T-19 cell infusion. Most patients (98.36%) received a Cy/Flu based regimen while only one patient (1.64%) received FLAG regimen. The CD19 CAR-T cells were infused at a dose of 5 ∗ 10^6^/kg (**Table 1**).

**Table 1:** Baseline characteristics in r/r B-ALL patients by severity of CRS^a^.

CRS Grade^b^	mCRS (0-2)	sCRS (3–4)	Total	Univariate Analysis P-Value	Multivariable Analysis P-Value
**Number of patients, n**	44	17	61		
**%**	72.13	27.87	100		
**Age, years**					
Median, [IQR]	30 [19.35,44.75]	34 [20.50,47.00]	32 [19.50,45.50]	0.540	
Range	9,73	9,68	9, 73		
**Sex, n (%)**					
Male	20 (66.67)	10 (33.33)	30 (49.18)	0.402	
Female	24 (77.42)	7 (22.58)	31 (50.82)		
**Genetic risk, n(%)**					
Poor	15 (55.56)	12 (44.44)	27 (44.26)	0.020*	0.025*
Good	29 (85.29)	5 (14.71)	34 (55.74)		
**Prior Lines of Therapy, n**					
Median, [IQR]	3.00 [2.00,4.75]	3 [2,4]	3 [2,4]	0.954	
Range	1,10	2,9	1,10		
**Numbers of relapses, n**					
Median, [IQR]	1 [1,1]	1 [1,1]	1 [1,1]	0.942	
Range	0,3	0,2	0,3		
**Prior Transplant, n (%)**					
Allogeneic	8 (66.67)	4 (33.33)	12 (19.67)	0.802	
Auto	1 (100)	0	1 (1.64)		
No	35 (72.92)	13 (27.08)	48 (78.69)		
**Lymphodepletion, n (%)**					
Cy/Flu based	43 (71.67)	17 (28.33)	60 (98.36)	1.000	
Non-Cy/Flu based	1 (100)	0	1 (1.64)		
**Response, n (%)**					
CR	37 (71.15)	15 (28.85)	52 (85.25)	0.995	
NR	7 (77.78)	2 (22.22)	9 (14.75)		
**ICANS, n (%)**					
Yes	0	3 (100%)	3 (4.92%)	0.019*	
No	44 (75.86%)	14 (24.14%)	58 (95.08%)		
**Marrow Disease Burden, %**					
Median, [IQR]	4.0 [1.5,16.63]	21.20 [10.50,53.75]	10 [2.00,30.25]	0.002*	0.026*
Range	0, 91	0, 83.5	0, 91		
**MRD, %**					
Median, [IQR]	2.31 [0.1,32.83]	24.2 [11.21,61.00]	9.52[0.27,49.14]	0.014*	0.843
Range	0, 99.8	0.01, 87.09	0, 99.8		

CRS, cytokine release syndrome; Cy, cyclophosphamide; CR, complete remission; Flu, fludarabine; IQR, Interquartile; mCRS, mild CRS; MRD, minimal residual disease, NR, no remission; sCRS, severe CRS; ssCAR-T-19, IL-6 knocking down CD19 chimeric antigen receptor T.

a The data for age conformed to a normal distribution, and a t-test was used; the data for prior lines of therapy, number of the relapse, marrow disease burden and MRD conformed to a non-normal distribution, and a Mann–Whitney U test was used; the data for prior transplant, ssCAR-T-19 cell dose, and lymphodepletion were analyzed by Fisher Exact test; the data for sex, genetic risk and response were analyzed by Chi-Square test.

b CRS was defined as mCRS if graded 0–2 and sCRS if graded 3–4.

^*^p-values <0.05.

### Response, duration of response, and overall survival

**Figure 1:**
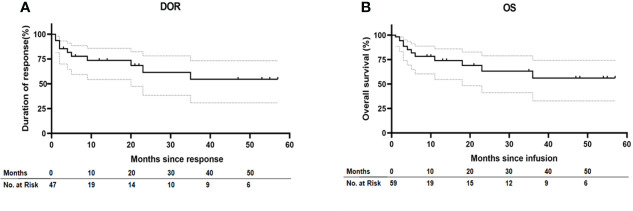
Duration of response and overall survival. **(A)** Kaplan–Meier estimates of the duration of response (DOR) in patients who achieve CR after ssCAR-T-19 infusion with censoring patients at subsequent allogeneic stem-cell transplant. **(B)** Kaplan–Meier estimate of overall survival (OS) in all the patients without censoring of patients at subsequent allogeneic stem-cell transplant. Median DOR in patients who achieved CR and median OS in all the patients were not reached. Dashed lines in **(A)** and **(B) **denote the 95% confidence interval.

Fifty-two patients (85.25%) achieved CR, while nine patients (14.75%) were considered NR (**Table 1**). The DOR among the patients who achieved CR at 36 months was 56.26% (32.81%–74.31%) and the median DOR was not reached among these patients with censoring patients at subsequent allogeneic stem-cell transplant. OS among all patients at 36 months was 54.72% (30.90%–73.38%) and the median OS was not reached in all patients without censoring of patients at subsequent allogeneic stem-cell transplant (**Figure 1**).

### Clinical description of CRS

Most patients (44 of 61, 72.13%) had either no CRS (grade 0), or grade 1 to 2 CRS, which was defined as mCRS, whereas 17 patients (27.87%) developed grade 3 to 4 CRS, which was defined as sCRS. No grade 5 CRS was reported among the enrolled 61 patients (**Table 1**). The median time of CRS beginning, peak CRS, and CRS remission were 2 days, 4 days, and 8 days after infusion in all r/r B-ALL patients, respectively (**Table 2**). Further analysis according to the CRS level showed that the median time of CRS beginning in patients with mCRS and sCRS was day 2 (IQR: day 1, 4) and day 1 (IQR: day 1, 2), respectively, and the difference was significant (*p* = 0.002). The median time of peak CRS in patients with mCRS and sCRS was similar; both were on day 4 but with different IQR (mCRS: day 2, 7 and sCRS: day 3, 9). The median time of CRS remission in patients with mCRS and sCRS was day 8 (IQR: day 6, 11.5) and day 8 (IQR: day 6, 10), respectively. sCRS could be resolved using tocilizumab or/and corticosteroids or/and ruxolitinib. The detailed medication is shown in **Supplementary Table 3**.

**Table 2:** Clinical description of CRS.

CRS Grade^a^	mCRS (1–2)	sCRS (3–4)	Total	Univariate Analysis P-Value^b^
**Number of patients, n**	33	17	50	
**%**	66	34	100	
**Onset time of CRS, days**				
Median, [IQR]	2 [1,4]	1 [1,2]	2 [1,3]	0.002^*^
Range	1,10	1,2	1,10	
**Time of peak CRS, days**				
Median, [IQR]	4 [2,7]	4 [3,9]	4 [3,7]	0.406
Range	1,10	2,14	1,14	
**Time of CRS remission, days**				
Median, [IQR]	8.00 [6.00,10.00]	8.00 [6.00,11.50]	8,00 [6.00,10.75]	0.665
Range	2, 19	3, 22	2, 22	
CRS treatment^c^, n (%)				
Corticosteroids only	4 (36.40%)	7 (63.60%)	11 (25.00%)	–
Tocilizumab only	0	1 (100%)	1 (2.27%)	
Ruxolitinib only	0	1 (100%)	1 (2.27%)	
Corticosteroids and Tocilizumab	0	2 (100%)	2 (4.55%)	
Corticosteroids and Ruxolitinib	0	2 (100%)	2 (4.55%)	
Without above treatment	27 (100%)	0	27 (61.36%)	

CRS, cytokine release syndrome; mCRS, mild CRS; sCRS, severe CRS.

a Eleven patients with grade 0 CRS were excluded to ensure the accuracy of the analysis results. CRS was defined as mCRS if graded 1–2 and sCRS if graded 3–4.

b The data for onset time of CRS, time of peak CRS and time of CRS remission were analyzed by Mann–Whitney U test.

c Six patients could not be assessed due to lack of CRS treatment information.

^*^p-values <0.05.

### Patient baseline characteristics associated with the development and severity of CRS

**Figure 2:**
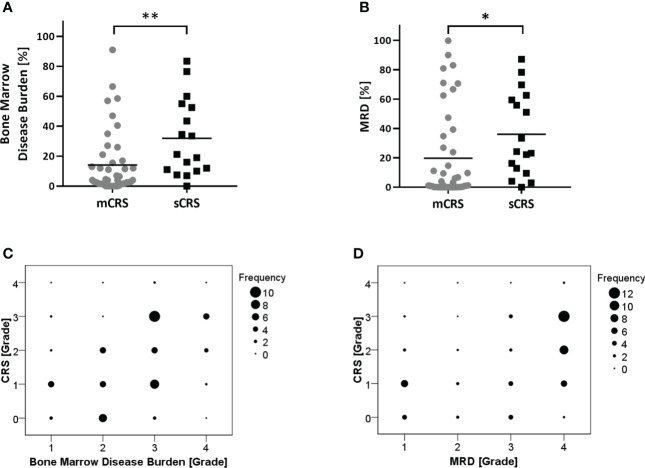
Analysis of patient characteristics associated with the severity of CRS. **(A, B)** Comparison of bone marrow disease burden or MRD between patients with mCRS and sCRS. All the patients were evaluated for bone marrow disease burden and MRD before ssCAR-T-19 cell infusion. Mean values were calculated for each group. *P*-values were calculated using Mann–Whitney U test. **means *p <*0.01, *p-values <0.05. **(C, D)** The test for linear trend between bone marrow disease burden or MRD and the severity of CRS. Bone marrow disease burden and MRD were divided into four groups and the CRS were graded into five levels. The size of the circle represented the number of patients. Mantel–Haenszel chi-square test was used to calculate the linear trend.

**Figure 3:**
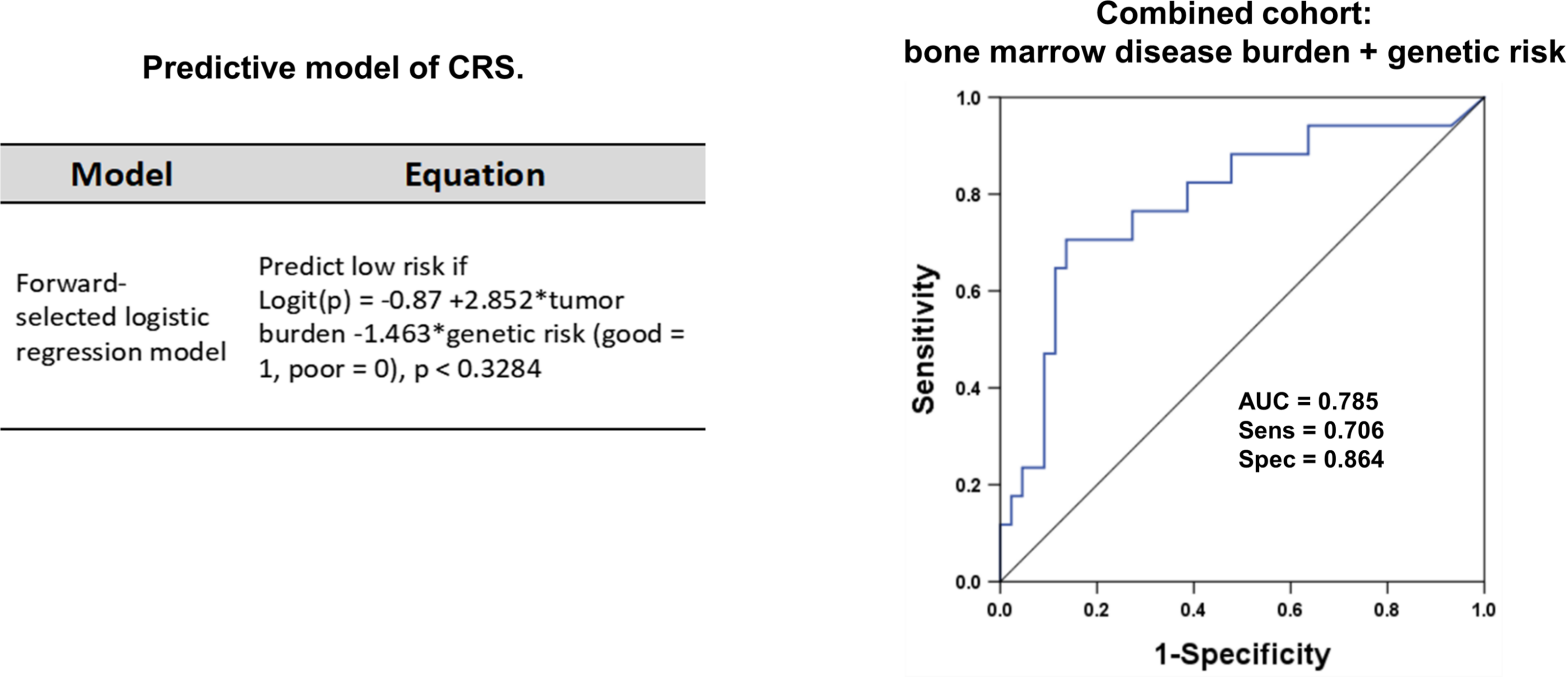
The ROC curve for the two-variable regression model. Sixty-one r/r B-ALL patients receiving ssCAR-T-19 therapy were enrolled in the forward-selected logistic regression model. The model was used to predict which patients would develop sCRS after ssCAR-T-19 cell infusion. The logit(p) function transformed the logistic regression score into the predicted probability of the case model. Logit (p) = ln (p/1 − p). The ROC curve was drawn using the logistic regression score. The severity of CRS was predicted using bone marrow disease burden and genetic risk of patients with r/r B-ALL before ssCAR-T-19 infusion. The sensitivity was 70.6%, the specificity was 86.4%, and AUC was 0.785.

To identify patients at high risk of developing sCRS before ssCAR-T-19 infusion, we performed univariate analyses of the impact of baseline clinical characteristics, including age, sex, prior treatment, genetic risks, number of relapse, bone marrow disease burden, MRD, and lymphodepletion regimen on CRS. Patients with poor genetic risk (*p* = 0.02), a higher bone marrow disease burden (*p* = 0.002), and a higher MRD in the bone marrow (*p* = 0.014) were at a higher risk of developing sCRS (**Table 1**). The bone marrow disease burden of patients who developed sCRS was significantly higher than that of patients who developed mCRS (mCRS vs. sCRS, 14.1% ± 21.03% vs. 31.92% ± 25.77%, *p <*0.01, **Figure 2A**). The MRD of sCRS patients was significantly higher than that of mCRS patients (mCRS vs. sCRS, 19.77% ± 30.35% vs. 36.03% ± 28.43%, *p <*0.05, **Figure 2B**). To further understand the correlation between the bone marrow disease burden or the MRD and CRS, the test for linear trend was performed. The bone marrow disease burden was classified into four grades: grade 1, <1%; grade 2, ≥1% and <5%; grade 3, ≥5% and <50%; and grade 4, ≥50%. Similarly, the MRD was also classified into four grades: grade 1, <0.1%; grade 2, ≥0.1% and <1%; grade 3, ≥1% and <10%; and grade 4, ≥10%. Most cases were located on the diagonal and a moderately positive linear correlation was confirmed between the bone marrow burden or the MRD and CRS (bone marrow disease burden vs. CRS, χ^2^ = 13.514, *p <*0.001, r = 0.475, *p <*0.001, **Figure 2C**; MRD vs. CRS, χ^2^ = 13.328, *p <*0.001, r = 0.471, *p <*0.001, **Figure 2D**). However, stepwise multivariable analysis showed that poor genetic risk (*p* = 0.025) and a higher marrow disease burden (*p* = 0.026) rather than a higher MRD were independently associated with the development of sCRS when compared to mCRS (**Table 1**). With the forward-selected logistic regression model, we could predict which patients developed sCRS using bone marrow disease burden and genetic risk with a sensitivity of 70.6%, a specificity of 86.4%, and an AUC of 0.785 (**Figure 3**).

### Post-infusion laboratory findings including peak cytokines, CRP, and ferritin

**Figure 4:**
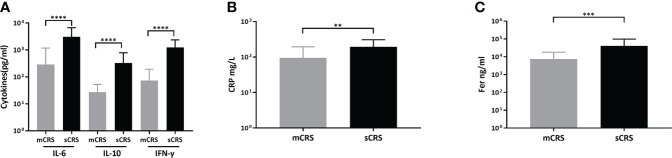
Peak cytokines, CRP, ferritin after ssCAR-T-19 infusion. **(A–C)** Levels of peak cytokines, CRP and ferritin of the patients after ssCAR-T-19 infusion. *P*-values were calculated using t-test. ****means *p*-values <0.0001, ***p <0.001, and **p <0.01. **(A)** IL6, IL10, and IFN-γ, **(B)** CRP and, **(C)** ferritin are shown in patients with mCRS and sCRS, respectively. The error bars in **(A–C)** represent mean ± SD.

After ssCAR-T-19 cell infusion, patients who developed sCRS exhibited significantly higher peak concentrations of IL-6 (mCRS vs. sCRS, 286.67 ± 896.34 pg/ml vs. 3,054.55 ± 3,698.03 pg/ml, *p <*0.001, **Figure 4A**, left), interleukin-10 (IL-10, mCRS vs. sCRS, 27.36 ± 25.69 pg/ml vs. 327.75 ± 461.96 pg/ml, *p <*0.001, **Figure 4A**, middle) and interferon-γ (IFN-γ, mCRS vs. sCRS, 73.94 ± 119.51 pg/ml vs. 1,253.65 ± 1,126.65 pg/ml, *p <*0.0001, **Figure 4A**, right) compared to the patients who developed mCRS. The peak CRP and ferritin of patients with sCRS were also higher than those of patients with mCRS (mCRS vs. sCRS, CRP: 95.60 ± 98.73 mg/L vs. 194.17 ± 114.29 mg/L, *p <*0.01, **Figure 4B**; ferritin, 7,618.04 ± 10,568.61 ng/ml vs. 40,981.33 ± 56,905.05 ng/ml, *p <*0.001, **Figure 4C**).

### Hematological and non-hematological side effects according to severity of CRS

**Figure 5:**
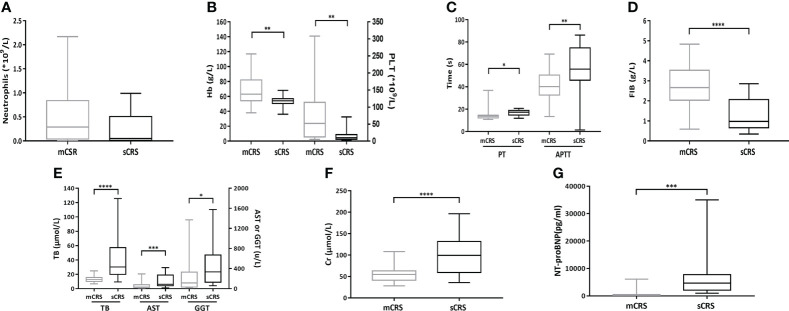
Hematologic and non-hematologic toxicities after ssCAR-T-19 infusion. **(A–G)** Hematologic and non-hematologic toxicities after ssCAR-T-19 infusion. *P*-values were calculated using t-test. ****means *p*-values <0.0001, ***p <0.001, **p <0.01, and *p <0.05. **(A)** Minimum absolute neutrophil count, **(B)** hemoglobin (Hb) and platelet count (PLT), **(C)** maximum PT and APTT, **(D)** minimum fibrinogen (FIB), **(E)** maximum total bilirubin (TB), serum aspartate aminotransferase (AST), and gamma-glutamyl transpeptidase (GGT), **(F)** creatinine (Cr), and **(G)** N-terminal pro-brain natriuretic peptide (NT-proBNP) is shown in patients with mCRS and sCRS, respectively. The upper and lower boundaries of each box indicate the 25th and 75th percentiles. The middle horizontal lines represent the median values and the whiskers mean the minimum and maximum.

The absolute neutrophil count (mCRS vs. sCRS: 0.48 ± 0.55 × 10^9^/L vs. 0.22 ± 0.32 × 10^9^/L, *p* = 0.08, **Figure 5A**) between the patients with mCRS and sCRS was similar while the hemoglobin (Hb, mCRS vs. sCRS: 69.30 ± 20.54 g/L vs. 53.41 ± 8.05 g/L, *p <*0.01, **Figure 5B**) and platelet (PLT, mCRS vs. sCRS: 77.48 ± 80.53 × 10^9^/L vs. 17.88 ± 20.45 × 10^9^/L, *p <*0.01, **Figure 5B**) were lower in patients with sCRS. Next, PT, APTT, and fibrinogen were examined in patients. Those with sCRS developed prolongation of the PT (mCRS vs. sCRS: 14.26 ± 4.16 s vs. 16.92 ± 2.50 s, *p <*0.05, **Figure 5C**) and APTT (mCRS vs. sCRS: 41.52 ± 12.91 s vs. 52.98 ± 22.09 s, *p <*0.01, **Figure 5C**) and falling fibrinogen concentrations (mCRS vs. sCRS: 2.77 ± 1.02 g/L vs. 1.26 ± 0.76 g/L, *p <*0.0001, **Figure 5D**). Moreover, the patients with sCRS exhibited elevated total bilirubin (TB, mCRS vs. sCRS: 13.38 ± 4.55 μmol/L vs. 42.08 ± 32.26 μmol/L, *p <*0.0001, **Figure 5E**, left), aspartate aminotransferase (AST, mCRS vs. sCRS: 56.47 ± 53.77 u/L vs. 155.08 ± 131.80 u/L, *p <*0.001, **Figure 5E**, middle) gamma-glutamyl transpeptidase (GGT, mCRS vs. sCRS: 206.27 ± 269.00 u/L vs. 463.17 ± 468.97 u/L, *p <*0.05, **Figure 5E**, right), creatinine (Cr, mCRS vs. sCRS: 54.18 ± 16.25 μmol/L vs. 102.20 ± 46.13 μmol/L, *p <*0.0001, **Figure 5F**), and N-terminal pro-brain natriuretic peptide (NT-proBNP, mCRS vs. sCRS: 880 ± 1,567 pg/ml vs. 6,686 ± 8,254 pg/ml, *p <*0.001, **Figure 5G**) when compared to those with mCRS.

### *In vivo* expansion and persistence of ssCAR-T-19 cell dynamics

**Figure 6:**
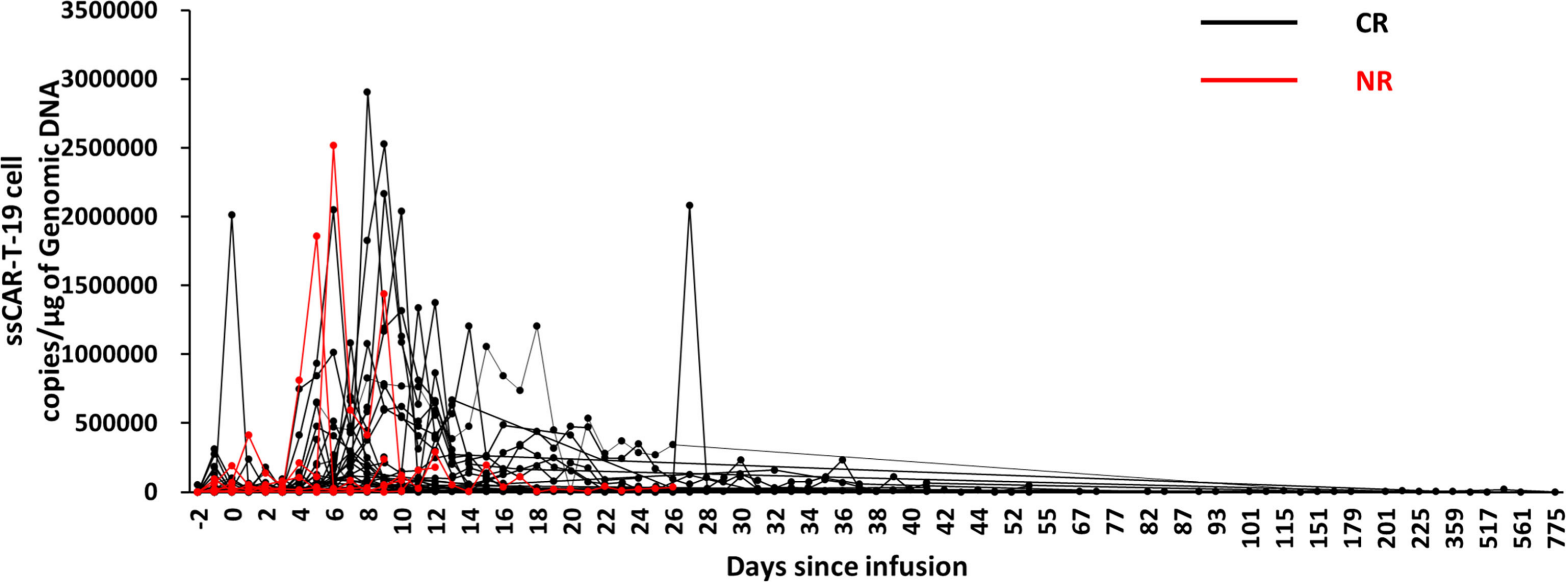
Expansion and persistence of ssCAR-T-19 cell in peripheral blood. The copies of ssCAR-T-19 cells in peripheral blood measured by qRT-PCR after infusion. Forty-eight patients with complete expansion data were included. Patients who achieved CR were shown in black while patients who achieved NR were shown in red.

After ssCAR-T-19 cell infusion, only one peak expansion could be observed in nine patients, while two or more expansion peaks were found in 39 patients. The maximum expansion occurred on day 8 (IQR: 5, 11.75) among all 48 patients with complete expansion data. The ssCAR-T-19 cell expansion in patients who achieved CR peaked at a median of 8 days (IQR: 5.75 to 12), while those who achieved NR peaked at 5.5 days (IQR: 1.75 to 10.5), and the difference was not significant. The median peak copies of ssCAR-T-19 cells in patients who achieved CR were 1.34 × 10^5^/μg (IQR: 5.37 × 10^4^/μg, 6.78 × 10^5^/μg) while those of non-responders were 1.87 × 10^5^/μg (IQR: 4.62 × 10^4^/μg, 2.02 × 10^6^/μg) without significant differences. In the CR group, CD19 CAR-T cells could be detected in seven of 42 patients beyond 100 days after infusion, among whom one had detectable genomic DNA after up to two years. This suggested the long-term persistence of ssCAR-T-19 cells in the patients after infusion (**Figure 6**).

### Disease burden, expansion of ssCAR-T-19 cells, response, and CRS

**Figure 7:**
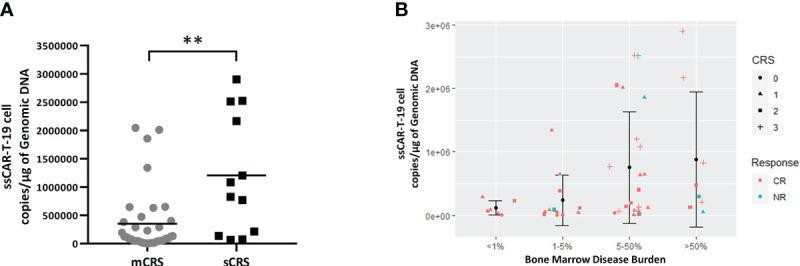
Relationship between peak copies of ssCAR-T-19 cell, bone marrow disease burden and CRS grade in r/r B-LL patients. **(A)** Comparison of ssCAR-T-19 cell expansion between patients with mCRS and sCRS. Mean values were calculated for each group. *P*-value was calculated using a Mann–Whitney U test. **means *p*-value <0.01. **(B)** Relationship between peak copies of ssCAR-T-19 cell, bone marrow disease burden, and CRS grade in r/r B-ALL patients. The bone marrow disease burden was divided into four groups. The y-axis represented the peak copies of the ssCAR-T-19 cells within 14 days after infusion. Mean values were calculated for each group and error bars indicate standard deviation. The different shapes of icons represented the grade of CRS. Red represented patients who achieved CR while blue represented patients who had no remission.

Next, we evaluated the relationships among disease burden, expansion of ssCAR-T-19 cells, response, and CRS grade. The expansion of ssCAR-19-T cells was much lower in patients with mCRS than that in patients with sCRS (mCRS vs. sCRS, 352,853 ± 563,924 copies/μg vs. 1,206,387 ± 1,058,071 copies/μg, *p <*0.01, **Figure 7A**). The bone marrow disease was stratified into four groups as above: <1%, ≥1% and <5%, ≥5% and <50%, and ≥50%. Although the differences were not significant, a trend of increased expansion was seen in patients with high disease burden. All patients with a disease burden of less than 1% achieved CR. No grade 3 CRS was observed in patients with a bone marrow disease burden of less than 5%. The analyses implied an association of more severe CRS in patients with a higher disease burden and larger expansion (**Figure 7B**).

## Discussion

The improved results of CD19 CAR-T-cell therapy recently brought hope to patients with relapsed or refractory B-cell malignancies. But as every rose has its thorn, alongside the impressive efficacy come the toxicities, of which the most common and dangerous are CRS and ICANS.

The essence of CAR-T therapy is targeted immunotherapy. The immune response and inflammatory response caused by cytokines released by CAR-T cells and other immune cells are the fundamental conditions for CAR-T cells to kill target cancer cells and achieve immunotherapeutic effects. However, excessive cytokine release, a cytokine storm, could lead to CRS. Previous studies indicated that the incidence of CRS was 55.3% and 95% in two pilot studies among both pediatric and adult patients (27, 28); 75% in 20 children and young adults with r/r B-ALL (29); 85% in 53 adults with relapsed B-ALL (30), and 80% in 25 pediatric/young adult patients with r/r B-ALL (31) in three phase I trials, respectively; 83% in 30 adults with B-ALL (32) in a phase I/II trial; and 77%–79% in pediatric and young adult patients with r/r B-ALL (14, 33) and 89% in 55 adult patients with r/r B-ALL (34) in three phase II multicenter trials, respectively. The incidence of sCRS in above studies was 13.2%–46%, and deaths occurred directly correlated with CRS (1.8%–3.8%) (16, 30, 32, 35).

The development of CRS, from onset to development to severe stage, is a dynamic process. There are three levels of strategies to deal with sCRS:

First of all, because CRS occurs and develops very rapidly in some patients, once sCRS occurs, it is necessary to passively administer drugs to control sCRS as soon as possible. The management of CRS by using tocilizumab proved to be efficient but does not appear to improve neurotoxicity because of its inability to pass the BBB and may even worsen it in some cases (20). Corticosteroids such as dexamethasone and cortisone might inhibit T-cell activation, proliferation, and eventually reduce CAR-T cell effectiveness, leading to the failure of CAR-T-cell therapy (22).

In addition to passively managing sCRS in the clinic, the second strategy is to design and develop new and safe CAR-T cells to reduce the incidence of sCRS, such as GM-CSF-deficient CAR-T cells through CRISPR/Cas9 disruption of GM-CSF during CAR-T-cell manufacture. But by far only preclinical data have been reported (36).

The interplay between CAR-T cells and tumor cells activates host bystander cells, especially monocytes/macrophages, eliciting a distortion of the cytokine network. Among the cytokines released by monocytes/macrophages and CAR-T cells, IL-6 plays a central role. An increasing number of studies indicate that monocyte and macrophage lineages are the key origins of IL-6 (37, 38). Notably, dendritic cells and even CAR-T cells are considered to participate in IL-6 production (38, 39). IL-6 released from CAR-T cells could trigger IL-6 secretion from monocytes (39). In a pre-clinical study, Tan et al. designed a CAR-T cell with a non-signaling membrane-bound IL-6 receptor (mbaIL-6) and found that mbaIL-6 expressed on the surface of T cells could rapidly remove IL-6 from the culture serum and circulation in a mouse model without affecting the anti-tumor potential of CAR-T cells (40). In a clinical trial, the researchers engineered an anti-CD19 or anti-BCMA (B-cell maturation antigen) CAR-T called CART-aIL6/IL1RA. This CAR-T product has been shown to reduce the incidence of IL-6 and IL-1-related CRS and ICANS by secreting anti-IL6 scFv and IL1 receptor antagonist (IL1RA), which could self-neutralize IL-6 and IL-1 in serum (41). Different from neutralizing IL-6 in the serum, we optimized the CD19 CAR-T cells by using a short hairpin RNA (shRNA) targeted at IL-6. We have successfully treated patients with relapsed B-ALL in the skin and testicles (42) and cerebral nervous system (CNS) (43) by applying ssCAR-T-19 cells and only grade 1 CRS was noted in these patients. Afterwards, we initiated this clinical study to explore the efficacy and safety of ssCAR-T-19 cells, which were designed as safer CAR-T products. Differences in study or trial designs, trial phases, CAR structures, patient populations, lymphodepletion regimens, and CAR-T-cell infusion doses present challenges in comparing results across our study and the above studies. In our study, CRS occurred in 81.97% of the enrolled 61 r/r B-ALL patients. Notably, the majority were mCRS in our study. No significant differences in the incidences of both mCRS and sCRS between patients who ≤25 years old and above 25 years old could be observed (**Supplementary Table 4**). In particular, in our study, grade 4 CRS was quite rare (1.64%) and no grade 5 ever happened (**Supplementary Table 5**). Thus, the ssCAR-T-19 cell therapy in our study was effective and safe to treat r/r B-ALL with a CR rate of 85.25% and sparse grade 4–5 CRS cases. In addition, both the median DOR and OS were not reached (more than 50 months). The outstanding therapeutic results in our studies may be related to the safe preference of CAR-T products we used, as well as the infusion strategy of the CAR-T cells using split doses, which was consistent with the report of Frey et al. that fractionated dosing of CTL019 improved the safety profiles without compromising efficacy in adults with r/r ALL (44).

The third strategy to deal with sCRS is to identify risk factors before CAR T-cell infusion that are associated with the incidence and severity of subsequent CRS to allow identification of patients who are at high risk of developing sCRS and might be candidates for early intervention studies. Tedesco et al. summarized the predictive biomarkers of CRS in their systematic review (45), including bone marrow blast, platelet, CRP, ferritin, and IFN-γ, and cytokines IL-2, L-6, IL-8, and IL-10. Similarly, our data demonstrated that IL6, IL-10, IFN-γ, CRP, and ferritin were significantly elevated and were higher in patients with sCRS. However, the levels and time-point of these biomarkers are important to differentiate sCRS. In addition, our data implied that sCRS (day 1) occurred earlier than mCRS (day 2). This is in line with the work of Hay KA et al. They performed classification-tree modeling and found that fever ≥38.9°C within 36 h of CAR T-cell infusion, a serum MCP-1 concentration ≥1,343.5 pg/ml enhanced identification of 4–5 CRS with a sensitivity of 100% and specificity of 95% (16). In our study, the patients with sCRS developed more severe hematological toxicity and liver, renal, and cardiac dysfunction compared with the patients with mCRS. Although these complications may be resolved by appropriate intervention, they alert clinicians to the dangers of cytokine release syndrome. Mitigating CRS may be the basis for solving these complications.

Moreover, our data demonstrated that larger ssCAR-T-19 cell expansion correlated with the severity of CRS. However, maximum expansion of ssCAR-T-19 cells was not involved in our predictive model because it was a post-infusion factor and occurred on a median day of day 8 (IQR: day 5, 12) when sCRS (day 1, IQR: day 1, 2) had already happened. In addition, patients with a higher bone marrow disease burden tended to result in greater ssCAR-T-19 expansion, and all the patients with a disease burden of less than 1% developed merely mCRS and achieved CR. The association of higher pretreatment tumor burden and sCRS has been proven in many studies (29, 46, 47). Therefore, the reduction of tumor burden prior to infusion cannot be overemphasized.

Good predictors should offer the clinician foresight before CRS happened rather than hindsight. Therefore, in the real world, the value of the maximum fold change or peak level of these biomarkers alone as an early predictor would be discounted. Except for the above biomarkers, our study assessed the readily available baseline metrics to roughly predict the severity of CRS. Univariate analysis and multivariable analysis identified that poor genetic risk and a higher marrow disease burden were independent factors associated with sCRS employing ssCAR-T-19 therapy. In our logistic regression model, the combination of bone marrow disease burden and genetic risk has 86.4% specificity and 70.6% sensitivity for sCRS with an AUC of 0.785. Previous studies have also demonstrated that a higher bone marrow blast correlated with sCRS (2, 48), and reducing tumor burden could decrease CRS. However, few previous studies have explored the influence of cytogenetic risk on CRS, merely listing the Ph-positive subtype. Our data indicated the importance of differentiating good or poor cytogenetic risk according to NCCN guidelines in order to identify sCRS. Our model was somehow less labor-intensive when applying limited baseline parameters but was low-hanging fruit when compared with the study of Teachey et al. (11). However, currently, the ideal predictor panel remains unclear since no predictive models have been validated universally. More clinical trials are warranted to identify the highly specific, early onset, and cost-effective predictors of sCRS.

In conclusion, ssCAR-T-19 cell therapy in our study induced fewer grade 4–5 CRS and baseline characteristics, namely high bone marrow disease burden and poor genetic risk before infusion, are independent risk factors of sCRS. The data from our study provide the clinicians with important high-risk factors of CRS and give them more time to manage the CRS actively before it deteriorates to sCRS by using these risk factors, fractional infusion, monitoring the cytokine levels of the patient, CAR-T-cell expansion, and clinical symptoms frequently.

## Data availability statement

The raw data supporting the conclusions of this article will be made available by the authors, without undue reservation.

## Ethics statement

The studies involving human participants were reviewed and approved by the Ethics Committee of the First Affiliated Hospital of Soochow University. Written informed consent to participate in this study was provided by the participants’ legal guardian/next of kin.

## Author contributions

S-LXue conceived, designed the clinical trial and edited manuscript. W-JGong and YQ collected, analyzed the data, and wrote the manuscript. M-HLi provided essential materials. L-YChen revised the manuscript. Y-YLi and J-QYu collected the data. L-QKang and LYu designed and discussed the clinical trial. A-NSun and D-PWu read the manuscript and gave comments. All authors contributed to the article and approved the submitted version.

## Funding

This work was supported by the grants from the National Natural Science Foundation of China (Grant No. 81970138), the Translational Research Grant of NCRCH (Grant No. 2020ZKMB05), the Jiangsu Province “333” Project, Social Development Project of the Science and Technology Department of Jiangsu (Grant No. BE2021649), and the Gusu Key Medical Talent Program (Grant No. GSWS2019007).

## Acknowledgments

This study acknowledged data from the First Affiliated Hospital of Soochow University and technical support from Shanghai Unicar-Therapy Bio-Medicine Technology Co., Ltd.

## Conflict of interest

Authors M-HLi, L-QKang, and LYu were employed by the Shanghai Unicar-Therapy Bio-Medicine Technology Co., Ltd.

The remaining authors declare that the research was conducted in the absence of any commercial or financial relationships that could be construed as a potential conflict of interest.

## Publisher’s note

All claims expressed in this article are solely those of the authors and do not necessarily represent those of their affiliated organizations, or those of the publisher, the editors and the reviewers. Any product that may be evaluated in this article, or claim that may be made by its manufacturer, is not guaranteed or endorsed by the publisher.

## Glossary

**Table:**

APTT	Activated partial thromboplastin time
Ara-C	Cytosine arabinoside
AST	Aspartate aminotransferase
BBB	Blood–brain barrier
CAR-T	Chimeric antigen receptor T
CNS	Cerebral nervous system
CR	Complete remission
Cr	Creatinine
CRP	C-reactive protein
CRS	Cytokine release syndrome
Cy	Cyclophosphamide
DOR	Duration of response
EMA	European Medicines Agency
FDA	Food and Drug Administration
Flu	Fludarabine
G-CSF	Granulocyte -colony stimulating factor
GGT	Gamma-glutamyl transpeptidase
GM-CSF	granulocyte-macrophage colony-stimulating factor
HB	Hemoglobin
HSCT	Hematopoietic stem cell transplantation
HSCT	Hematopoietic stem cell transplantation
HSCT	Hematopoietic stem cell transplantation
ICANS	Immune effector cell associated neurotoxicity syndrome
IFN-γ	Interferon-γ
IL-10	Interleukin-10
IL-6	Interleukin-6
IQR	Interquartile
MCP-1	Monocyte chemoattractant protein 1
mCRS	Mild CRS
MRD	Minimal residual disease
NR	No remission
NT-proBNP	N-terminal pro-brain natriuretic peptide
ORR	Overall response rate
OS	Overall survival
PLT	Platelet
PR	Partial remission
PT	Prothrombin time
r/r B-ALL	Relapsed or refractory B-cell acute lymphoblastic leukemia
r/r DLBCL	Relapsed or refractory diffuse large B-cell lymphoma
r/r MCL	Relapsed or refractory mantle-cell lymphoma
scFv	Single chain variable fragment
sCRS	Severe CRS
SD	Standard deviation
shRNA	Short hairpin RNA
ssCAR-T-19	IL-6 knocking down CD19 CAR-T
TB	Total bilirubin
