# Supplementary material for: Investigation of the risk factors to predict cytokine release syndrome in relapsed or refractory B-cell acute lymphoblastic leukemia patients receiving IL-6 knocking down anti-CD19 chimeric antigen receptor T-cell therapy
Source: Front Immunol. 2022 Aug 29;13:922212. doi: 10.3389/fimmu.2022.922212 (PMC9464804; doi:10.3389/fimmu.2022.922212)
Supplement: Supplementary file 1 [file DataSheet_1.docx]

Supplementary Material

**SUPPLEMENTARY TABLE 1. Cytogenetic risk for B-ALL**

| **Risk** | **Cytogenetics** |
| --- | --- |
| **Good** | Hyperdiploidy (51-65 chromosomes) |
|  | - Cases with trisomy of chromosomes 4, 10, and 17 appear to have the most favorable outcome |
|  | t(12;21)(p13;q22): ETV6-RUNX1 |
| **Poor** | Hypodiploidy(<44 chromosomes)  KMT2A rearranged (t[4;11] or others)  t(v;14q32)/IgH  t(9;22)(q34;q11.2): BCR-ABL1 (defined as high risk in the pre-TKI era)  Complex karyotype (5 or more chromosomal abnormalities)  BCR-ABL1-like (Ph-like) ALL   - JAK-STAT (CRLF2r,d,c EPORr, JAK1/2/3r, TYK2r, mutations of SH2B3, IL7R, JAK1/2/3) - ABL class (rearrangements of ABL1, ABL2, PDGFRA, PDGFRB, FGFR) - Other (NTRKr, FLT3r, LYNr, PTL2Br)   Intrachromosomal amplification of chromosome 21 (iAMP21)  t(17;19): TCF3-HLF fusion  Alterations of IKZF1 |

*Cited in NCCN Guidelines (version 1.2021)

**SUPPLEMENTARY TABLE 2. CRS Grading System**

| **CRS Grading** | **Grade 1** | **Grade 2** | **Grade 3** | **Grade 4** | **Grade 5** |
| --- | --- | --- | --- | --- | --- |
| **Temperature** | Fever≥ 38.0℃ | High fever for more than 5 consecutive days |  |  | Death due to CRS in  which another cause is not the principle factor leading to this outcome. |
| **Circulation** |  | Hypotension, but can be corrected by rehydration | Hypotension that can only be corrected with antihypertensive drugs |  |  |
| **Respiration** |  | Hypoxia: but can be corrected by <40% (7L/min) oxygen | Hypoxia: but can be corrected by <40% (7L/min) oxygen | Ventilator required |  |
| **Consciousness** |  |  | Neurologic dysfunction(Including altered confusion, retardation and seizures） | Disorders of consciousness and persistent seizures |  |
| **Organ dysfunction** |  | Grade 2 | Grade 3-4 | Grade 4 |  |
| **Inflammatory indicators** | CRP less than 10-fold increase | 10-fold increase < CRP < 25-fold increase  50-fold increase < IL-6 < 250-fold increase | 25-fold increase < CRP< 80-fold increase  250-fold increase < IL-6< 500-fold increase |  |  |

*Modified from NCCN Guidelines (version 1.2021) and CTCAE version 5.0

**SUPPLEMENTARY TABLE 3. Treatment of CRS.**

| **CRS Grade** | **Patient** | **Dexamethasone/mg** | **Methylprednisolone/mg** | **Tocilizumab/mg** | **Ruxolitinib/mg** |
| --- | --- | --- | --- | --- | --- |
| **Grade 1** | Patient 1 | 5 | 0 | 0 | 0 |
|  | Patient 2 | 5 | 0 | 0 | 0 |
| **Grade 2** | Patient 3 | 30 | 0 | 0 | 0 |
|  | Patient 4 | 5 | 0 | 0 | 0 |
| **Grade 3** | Patient 5 | 135 | 0 | 0 | 160 |
|  | Patient 6 | 0 | 400 | 0 | 0 |
|  | Patient 7 | 0 | 0 | 320 | 0 |
|  | patient 8 | 20 | 0 | 0 | 0 |
|  | patient 9 | 0 | 40 | 720 | 0 |
|  | patient 10 | 120 | 0 | 0 | 0 |
|  | Patient 11 | 5 | 40 | 0 | 0 |
|  | Patient 12 | 120 | 0 | 0 | 0 |
|  | Patient 13 | 0 | 0 | 0 | 350 |
|  | Patient 14 | 0 | 40 | 0 | 0 |
|  | Patient 15 | 5 | 0 | 0 | 570 |
|  | Patient 16 | 80 | 15 | 0 | 0 |
| **Grade 4** | Patient 17 | 80 | 0 | 400 | 0 |

Abbreviations: CRS = cytokine release syndrome.

Grade 1-4 CRS occurred in 50 patients. 1 patient with grade 1 CRS, 1 patient with grade 2 CRS and 4 patients with grade 3 CRS lacked treatment information of CRS. 4 patients with grade 1-2 CRS only received corticosteroids, 13 patients with grade 3-4 CRS received corticosteroids or/and tocilizumab or/and ruxolitinib. 15 patients with grade 1 CRS and 12 patients with grade 2 CRS did not receive special treatment of the above medications.

**SUPPLEMENTARY TABLE 4. Incidence of CRS according to the grade and age**

| **CRS Grade** | **mCRS(0-2)** | **sCRS(3-4)** | **Total** |
| --- | --- | --- | --- |
| **Age, years** |  |  |  |
| **≤25, n, %** | 16(72.73) | 6(27.27) | 22(100) |
| **>25, n, %** | 28(71.79) | 11(28.21) | 39(100) |

Abbreviations: CRS = cytokine release syndrome

**SUPPLEMENTARY TABLE 5. Incidence of CRS according to the grade**

| **CRS Grade** | **0** | **1** | **2** | **3** | **4** | **Total** |
| --- | --- | --- | --- | --- | --- | --- |
| **Number of patients, n** | 11 | 19 | 14 | 16 | 1 | 61 |
| **%** | 18.03 | 31.15 | 22.95 | 26.23 | 1.64 | 100 |

Abbreviations: CRS = cytokine release syndrome


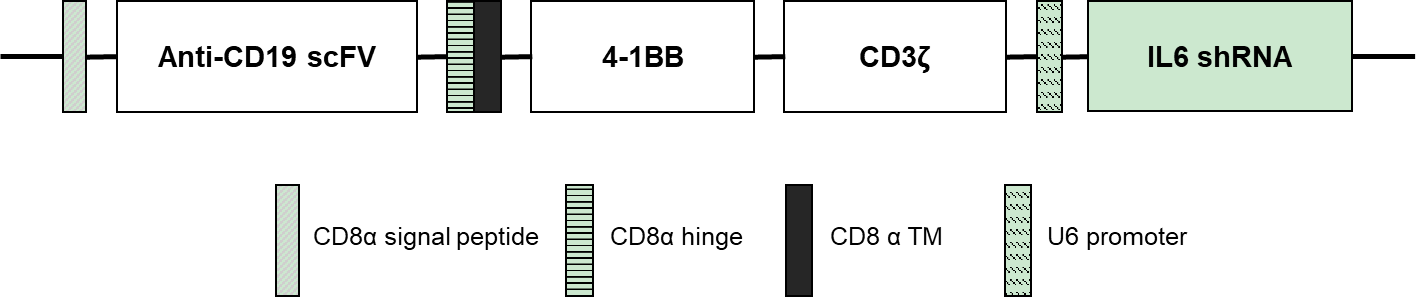


**SUPPLEMENTARY FIGURE 1.** **The structure of the recombinant lentiviral vectors of ssCAR-T-19 cells.** The recombinant lentiviral vectors contained an anti-CD19 single chain variable fragment (scFv), a 4-1BB costimulatory moiety and a CD3zeta activation domain with an IL-6 shRNA element against IL-6.
